# A Noninvasive Approach to Assess the Prevalence of and Factors Associated With Anemia Risk in Malaysian Children Under Three Years of Age: Cross-Sectional Study

**DOI:** 10.2196/58586

**Published:** 2025-03-24

**Authors:** Muhammad Yazid Jalaludin, Ho Bee Kiau, Suriati Hasim, Wai Khew Lee, Angie Low, Nik Harlina Nik Kazim, Jia Tse Hoi, Sri Wahyu Taher

**Affiliations:** 1 Faculty of Medicine, University Malaya, Lembah Pantai, Kuala Lumpur, 59100, Malaysia, 60 79492065, 60 79494704; 2 Klinik Kesihatan Bandar Botanik, Selangor, Malaysia; 3 Klinik Kesihatan Endau, Johor, Malaysia; 4 Klink Kesihatan Luyang, Sabah, Malaysia; 5 Danone Specialized Nutrition (Malaysia), Kuala Lumpur, Malaysia; 6 Klinik Kesihatan Wakaf Bahru, Kelantan, Malaysia; 7 Danone Research & Innovation, Singapore, Singapore; 8 Klinik Kesihatan Simpang Kuala Kedah, Kedah, Malaysia

**Keywords:** anemia, iron deficiency, children, Masimo Rad-67, noninvasive assessment, Malaysia

## Abstract

**Background:**

Anemia remains a significant public health concern with adverse effects among children. Noninvasive screening assessments enable the early detection and prompt treatment of anemia. However, there is limited literature on the use of such screening assessments.

**Objective:**

The study aimed to assess the prevalence of and factors associated with being at risk of anemia among Malaysian children aged ≥6 months to ≤36 months by using a noninvasive hemoglobin assessment.

**Methods:**

This was a cross-sectional study (from July to December 2022) of outpatient Malaysian children, aged ≥6 months to ≤36 months, who were selected from five maternal-and-child health clinics by convenience sampling. At risk of anemia was defined as a total hemoglobin level of <12 g/dL, measured using the Masimo Rad-67, a noninvasive screening device for total hemoglobin levels. The *χ*^2^ and multiple logistic regression analyses were used to assess the prevalence and factors associated with being at risk of anemia, using R-Studio (version 4.0.0).

**Results:**

The study included 1201 participants, of whom 30% (95% CI 28‐33) were at risk of anemia. Children aged 6‐12 months (210/364, 57.7%, *P*<.001), those of Asian Malay race (238/364, 65.4%, *P*<.05), those residing in the Klang district (123/371, 33.9%, *P*<.05), those born via a normal vaginal delivery (275/364, 75.5%, *P*<.05), those without a family history of thalassemia (284/364, 78.0%, *P*<.05), and those with lower weight-for-age Z scores (*P*<.05) were associated with being at risk of anemia. Children aged 6‐12 months (adjusted odds ratio=1.73; 95% CI 1.34‐2.24) had higher odds of being at risk of anemia compared to children aged >12‐36 months. However, weight-for-age (adjusted odds ratio=0.88; 95% CI 0.80‐0.98) was associated with lower odds of being at risk of anemia.

**Conclusions:**

The current study revealed a substantial prevalence of Malaysian children being at risk of developing anemia. The study results therefore imply a need for more community education and awareness on anemia, including nutrition education, as well as targeted community screening to enable the early detection and prompt treatment of anemia cases. Anemia reduction strategies in Malaysia should consider the highlighted factors indicative of higher risk of anemia.

## Introduction

Anemia is a specific condition where the body does not have enough normal or healthy red blood cells or hemoglobin (Hb) to provide adequate oxygen to the body tissues. It is usually caused by iron deficiency, which is the most common micronutrient deficiency in both low-income and high-income countries [1,2]. Generally, it takes at least several weeks after the depletion of iron stores before anemia develops. When iron deficiency occurs, Hb concentrations are reduced to below-optimal levels, a condition known as iron deficiency anemia (IDA) [2], which is the most common type of anemia found in children. Among children, the most common causes of IDA include insufficient iron intake along with rapid growth, low birth weight, and gastrointestinal losses, among others [3,4].

Generally, the prevalence of anemia and IDA in low-income countries is three to four times higher than that in high-income countries [1]. The global prevalence of anemia in 2019 was 39.8% in children aged 6‐59 months, with 269 million children having anemia, while in Malaysia, the prevalence of anemia was 24.6% in children of the same age [5]. In Malaysia, the current prevalence of anemia is approximately 46.5% among children, and 1 in 3 children (<5 years of age) has iron deficiency [6,7].

Iron deficiency can occur without anemia; this occurs when the iron store is depleted while the individual is still having normal Hb levels. IDA is a situation where the iron store and Hb levels are both below normal levels. Iron deficiency and IDA are typically diagnosed through an invasive blood test, with one of the diagnostic criteria for anemia being a Hb level below 11 g/dL [2]. The American Academy of Pediatrics recommends that all infants be tested for anemia starting between ages 9 months and 12 months [8], and that for those who have risk factors for iron deficiency, additional screenings will be required at later ages between 1 and 5 years [9].

Iron deficiency in childhood is associated with adverse outcomes such as impaired neurocognitive function and brain development, as well as compromised immune function [10,11]. In Malaysia, recent evidence shows that anemia is associated with cognitive and motor delays among infants aged 6‐12 months [12]. This implies that prompt diagnosis and anemia prevention are essential through early screening during infancy and early childhood.

The conventional diagnosis of anemia and IDA in infants is difficult, as blood sampling and obtaining sufficient blood volume are often difficult for typical laboratory detection. Moreover, these tests can be painful for the participant, expose the staff to human blood, and require training and quality control to ensure appropriate utilization and adherence to standards [3,11]. In this regard, an alternative device can be preferable, such as noninvasive Hb screening using a sensor that shines multiwavelengths of light through the finger of the patient [13]. Such noninvasive device options include the Masimo Pronto (Masimo Corporation) and Masimo Rad-67™ Pulse CO-Oximeters (Masimo Corporation). These devices were cleared by the US Food and Drug Authority for use in clinical and nonclinical settings to measure oxygen saturation [14] and have been used as a noninvasive measurement method for determining the Hb levels in children [15]. The Masimo Pronto and Masimo Rad-67™ Pulse CO-Oximeters have good accuracy and validity compared to Hemocue (HemoCue); however, the Hb levels were underreported for the devices compared to the levels determined in venous blood samples [16-18]. This study used the Masimo Rad-67™ Pulse CO-Oximeter for anemia screening.

This study aimed to assess the prevalence and factors associated with being at risk of anemia (defined as total Hb levels <12 g/dL) among Malaysian children aged ≥6 to ≤36 months by using noninvasive Hb assessment (the Masimo Rad-67 Pulse CO-Oximeter). We used a noninvasive approach for anemia screening and compared the prevalence rates with those obtained in previous studies. Using a noninvasive screening approach offers the potential for the early detection and prompt treatment before worsening of the child’s condition to severe anemia-related complications.

## Materials and Methods

### Study Design

This was a cross-sectional study among children aged ≥6 months to ≤36 months conducted in five maternal-and-child health clinics and Ministry of Health primary care clinic settings in urban and rural areas of Malaysia from July to December 2022. The clinics were chosen and the study participants were enrolled based on the need to include major ethnic groups in Malaysia in the study via convenience sampling.

### Participants and Sample Size Planning

The study participants were outpatient children aged ≥6 months to ≤36 months at maternal-and-child health clinics accompanied by their primary caretakers (ie, parents, grandparents, or relatives) who were aged ≥18 years and could speak English, Bahasa Malaysia, or Chinese (Mandarin). Children who came to the clinics for routine vaccinations or routine health examinations were enrolled if the study requirements were met. The plan was to have 50% of participants aged 6‐12 months and 50% aged >12‐36 months, with the following ethnicity distribution: 69.8% Malay and Bumiputera, 22.4% Chinese, 6.8% Indian, and 1% others to ensure good representation of data from each site.

The study excluded children with any medical conditions for which interventions to increase nutritional intake might not be effective, per the investigator, in improving weight gain and the nutritional status. In addition, children participating in other studies involving iron-fortified foods or supplementation and those whose caretakers were not able to communicate effectively with the interviewers were excluded. The enrolment at the clinic was stopped when the participant number for specific sexes, age groups, and ethnicities, determined at the start of the study, was achieved. The World Health Organization and World Bank estimated the prevalence of anemia (ie, Hb level <11 g/dL) at 25% in 2019 among Malaysian children under 5 years of age [19]. In this study, we estimated that 30% of children aged ≥6 to ≤36 months will be at risk of anemia (ie, Hb level <12 g/dL), and considering a precision of ±3% and 95% confidence level, a sample size of 1200 subjects was required after accounting for 10% each of screen failures and dropouts. Any withdrawn subjects and screen failures were replaced until the sample size was obtained.

### Data Collection

The study was conducted in five maternal-and-child health clinics that were conveniently selected based on the research need: three from urban areas and two from rural areas of Malaysia. From each of the clinics, approximately 240 participants were selected. Interviewers were trained on the protocol. The trained interviewers identified and approached potential participants and asked the parents or guardians to participate in the survey. Those who were willing to participate were screened for eligibility, and written consent was obtained after they were briefed about the study objectives, procedures, and other details. Respondents were reminded of their right to quit the study at any time without any consequences, and anonymity was guaranteed.

During the assessment, a clip with a sensor attached to the Masimo Rad-67 Pulse CO-Oximeter was placed on the child’s finger or toe, and the reading took approximately 30 seconds. Children found to be at risk of anemia were referred to healthcare professionals for further clinical assessment. In addition, the child’s weight (kg) and length or height (cm) were measured and input into the Iron Strong app (Groupe Danone).

Data were collected via the Iron Strong app device, a data collection tool with an optical character recognition model that allows the user (by taking a picture) to accurately predict all units and digits on the Masimo Rad-67 Pulse CO-Oximeter and stores the data within the app or server during face-to-face interviews in English, Bahasa Malaysia, or Chinese (Mandarin). The interview and data collection took about 30‐40 minutes to complete.

### Measurements

#### Development of the Questionnaire

The questionnaire was developed by a panel of three public health researchers. Bilingual researchers translated the English version of the questionnaire into Bahasa Malaysia and Chinese (Mandarin). The agreed versions were back-translated into English by independent bilingual researchers to ensure linguistic equivalence. The questionnaire was not pretested because it mainly asked about respondents’ sociodemographic characteristics, anthropometric measurements, and 24-hour dietary recall.

#### Sociodemographic Characteristics

Participants reported their sociodemographic characteristics, such as the child’s sex, age, race, residence location, physical activity, as well as caregiver’s education level and household income. Birth history key indicators were asked, including mode of delivery, gestational age at birth, birth order, and the number of siblings. In addition, key indicators of family history such as thalassemia diagnosis and family history of anemia were asked.

#### Hb Level and Other Rad-67 Measurements

Anemia was assessed on the basis of the continuous total Hb level, with a cutoff value determined at <12 g/dL; this value was indicative of at risk of anemia, after considering the underestimation of the Hb concentration reported for Masimo devices [16-18]. This was measured using the Masimo Rad-67 Pulse CO-Oximeter, which is a noninvasive device for screening the total Hb levels. The reliability of the Masimo Rad-67 Pulse CO-Oximeter was reported to be ±1 g/dL versus the laboratory reference device in adults who had no motion, children, and infants. These findings have been validated by a group of researchers from Thailand [20].

#### Growth Parameters

Growth parameters were taken from the documentation available in the infant and child health record book. These parameters were taken by trained clinic nurses using standard methods.

#### Statistical Analysis

Categorical variables were summarized as the frequency and percentages, while continuous variables were summarized as the mean and standard deviation. Being at risk of anemia was the dependent variable. The *χ*^2^ test was used to test for the differences in background and predictor variables between children at risk of anemia and those not at risk. Bivariable and multivariable logistic regression models were fitted to explore the association between independent variables and the dependent variable (at risk of anemia). The multivariable regression models included significant factors on bivariable analysis as well as other factors known to be associated with being at risk of anemia from previous studies, regardless of their significance on bivariable analysis. We presented the respective crude odds ratios, adjusted odds ratios, 95% CI, and *P* values. All the analyses were performed using R-Studio (Posit, PBC), version 4.0.0, with a *P* value <.05 being the level of statistical significance.

### Ethical Considerations

The study was approved by the Medical Research and Ethics Committee, Ministry of Health Malaysia (Ref: 21‐02114-PV7(2)) and the Medical Research Ethics Committee, University of Malaya Medical Centre (Ref: 20211014‐10694). Study data were anonymized to ensure the privacy of all participants and were accessed only by authorized personnel. Informed consent was obtained from all participants prior to their involvement in the study. Participants were provided with detailed information about the study and were informed that participation was voluntary and that they could withdraw at any time without penalty. For secondary analyses of previously collected data, the original informed consent covered subsequent research uses, as confirmed by the ethics committee. Participants in the study received RM 20 (US $4.50) as monetary payment that was approved by the ethics board for their time and effort involved in participation.

## Results

**Figure 1.**
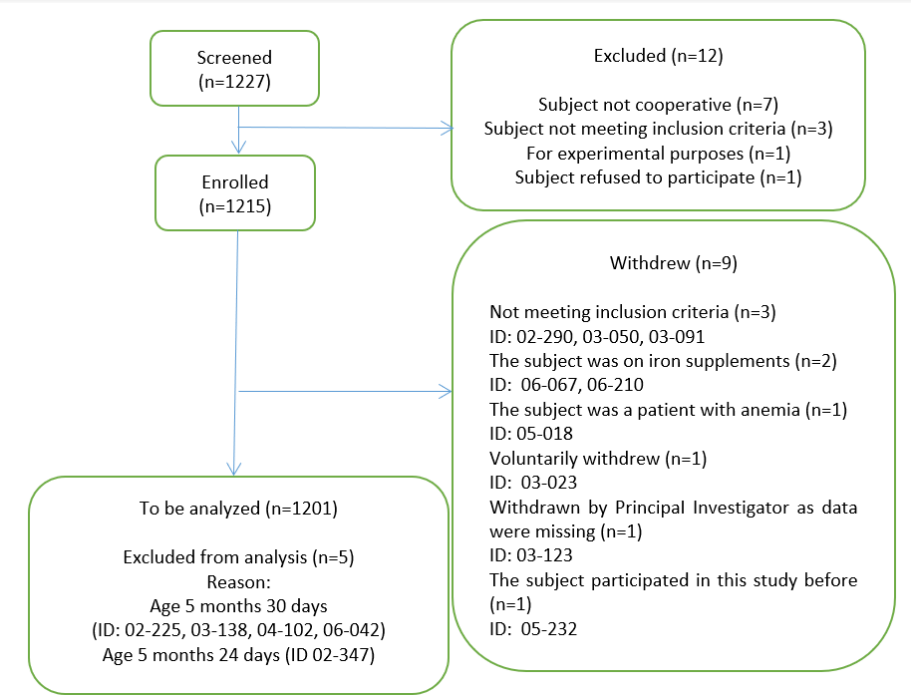
Flowchart depicting the selection of study participants for a cross-sectional study on the prevalence and risk factors of anemia among Malaysian children aged 6‐36 months. The study was conducted across five maternal-and-child health clinics in Malaysia between July and December 2022.

A total of 1227 potential respondents were reached, of whom 27 declined to participate due to various reasons, yielding a response rate of 97.9% (Figure 1).

### Characteristics of Participants

A total of 1,201 participants were included in this study (Table 1). Slightly more than half were male (631/1201, 52.5%) and aged >12‐36 months (610/1201, 51%); the majority were Asian Malay (727/1201, 60.5%) and from urban areas (687/1201, 57.2%). The majority had mothers with no university degree (898/1201, 74.8%), were born full-term (916/1201, 76%), via normal vaginal delivery (860/1201, 72%), and were of second or higher birth order (767/1201, 63.9%). Moreover, a very small number reported a family history of thalassemia (6/1201, 0.5%) with a quarter not knowing their thalassemia status (316/1201, 26.3%), while a majority had no family history of anemia (1078/1201, 89.8%) and reported moderate or vigorous physical activity (862/1201, 71.8%).

**Table 1.** Baseline characteristics of the study participants (N=1201).

Characteristics	Frequency, n (%)
Sex	
Female	570 (47.5)
Male	631 (52.5)
Age	
>12‐36 months	610 (51)
6‐12 months	591 (49)
Race	
Asian, Chinese	270 (22.5)
Asian, Bumiputera	119 (9.9)
Asian, Indian	85 (7.1)
Asian, Malay	727 (60.5)
Town or District	
Alor Setar	205 (17.1)
Klang	369 (30.8)
Endau	240 (20.0)
Kota Kinabalu	133 (11.1)
Tumpat	253 (21.1)
Residence location	
Rural	514 (42.8)
Urban	687 (57.2)
Mother’s education	
Bachelor’s degree or higher	303 (25.2)
Secondary education and below	898 (74.8)
Household income	
Low	585 (48.7)
Middle or high	616 (51.3)
Physical activity level	
Sedentary or lightly active	177 (14.7)
Moderately active or vigorously active	862 (71.8)
No activity recorded	162 (13.5)
Gestational age at birth	
Term	916 (76)
Preterm	285 (24)
Mode of delivery	
Vaginal	860 (72)
Cesarean	341 (28)
Birth order	
First	434 (36.1)
Second or more	767 (63.9)
Family history of thalassemia	
Yes	6 (0.5)
No	879 (73.2)
Not known	316 (26.3)
Family history of anemia	
Yes	123 (10.2)
Not known	1078 (89.8)

### Population at Risk of Anemia and Other Masimo Rad-67 Pulse CO-OximeterParameters

Of the 1,201 participants, 364 (30%, 95% CI 28‐33) were identified as being at risk of anemia on the Masimo Rad-67 Pulse CO-Oximeter (Table 2). Additionally, participants had a mean (SD) weight-for-age Z score of −0.67 (1.29), length-for-age Z score of −0.65 (1.72), and weight-for-length Z score of −0.41 (1.29).

**Table 2.** Population at risk of anemia and other Rad-67 measurements (N=1201).

Study site	Frequency of subjects at risk of anemia, n/N (%)
Alor Setar	39/205 (10.7)
Klang	123/371 (33.9)
Endau	60/240 (16.5)
Kota Kinabalu	43/133 (11.8)
Tumpat	98/252 (27.0)

### Distribution of Being at Risk of Anemia by Study Variables

The distribution of being at risk of anemia across the study variables is shown in Table 3. Children aged 6‐12 months (n= 210, 57.7%), those of Asian Malay race (n=238, 65.4%), those from Klang district (n=123, 33.9%), those birthed via normal vaginal delivery (n=275, 75.5%), and those without a history of thalassemia (n=284, 78.0%) were associated with being at risk of anemia. Additionally, children at risk of anemia had significantly lower weight-for-age Z scores (ie, a greater negative deviation from the normal weight) than those at no risk.

**Table 3.** Distribution of being at risk of anemia across study variables among Malaysian children aged ≥6 months to ≤36 months.

Variable	Not at risk of anemia, N=837	At risk of anemia, N=364	*P* value
Sex, n (%)			.87
Female	396 (47.3)	174 (47.8)	
Male	441 (52.7)	190 (52.2)	
Age, n (%)			<.001
>12‐36 months	456 (54.5)	154 (42.3)	
6‐12 months	381 (45.5)	210 (57.7)	
Race, n (%)			.02
Asian, Chinese	201 (24.0)	69 (19.0)	
Asian, Bumiputera	79 (9.4)	40 (11.0)	
Asian, Indian	68 (8.1)	17 (4.7)	
Asian, Malay	489 (58.4)	238 (65.4)	
Residence location, n (%)			.19
Rural	348 (41.6)	166 (45.6)	
Urban	489 (58.4)	198 (54.4)	
Education, n (%)			.75
Bachelor’s degree or higher	209 (25.0)	94 (25.8)	
Secondary education and below	628 (75.0)	270 (74.2)	
Household income, n (%)			.08
Low	394 (47.1)	191 (52.5)	
Middle or high	443 (52.9)	173 (47.5)	
Gestational age at birth, n (%)			.83
Term	637 (76.1)	279 (76.6)	
Preterm	200 (23.9)	85 (23.4)	
Mode of delivery, n (%)			.046
Vaginal	585 (69.9)	275 (75.5)	
Cesarean	252 (30.1)	89 (24.5)	
Birth order, n (%)			.17
First	313 (37.4)	121 (33.2)	
Second or more	524 (62.6)	243 (66.8)	
Siblings, n (%)			.37
No	289 (34.5)	116 (31.9)	
Yes	548 (65.5)	248 (68.1)	
Family history of thalassemia, n (%)			.03
Yes	4 (0.5)	2 (0.5)	
No	595 (71.1)	284 (78.0)	
Not known	238 (28.4)	78 (21.4)	
Family history of anemia, n (%)			.57
Yes	83 (9.9)	40 (11.0)	
Not known	754 (90.1)	324 (89.0)	
Weight-for-age Z score, mean (SD)	−0.62 (1.35)	−0.78 (1.15)	.03
Length-for-age Z score, mean (SD)	−0.60 (1.77)	−0.77 (1.57)	.10
Weight-for-length Z score, mean (SD)	−0.38 (1.36)	−0.47 (1.09)	.20

### Factors Associated With Being at Risk of Anemia Among Malaysian Children Aged ≥6 Months to ≤36 Months

The results of bivariable and multivariable logistic regression are detailed in Table 4. In our logistic regression analysis examining being at risk of anemia in children, the primary independent variable, weight-for-age Z score, showed a statistically significant inverse association with being at risk of anemia both in univariable (crude odds ratio=0.91, 95% CI 0.82‐1.00; *P*=.046) and multivariable models (adjusted odds ratio=0.88, 95% CI 0.80‐0.98; *P*=.020), indicating that higher Z scores were associated with reduced odds of being at risk of anemia. Among the covariates, only age showed a significant association; specifically, children aged 6‐12 months had 1.73 times higher odds of being at risk of anemia compared to those aged 12‐36 months (adjusted odds ratio=1.73, 95% CI 1.34‐2.24; *P*<.001). Other demographic and medical factors, such as sex, race, residence location, and socioeconomic status, did not significantly impact being at risk of anemia (Table 4). This study underscores the importance of weight monitoring and age-specific interventions in mitigating anemia risk in children.

**Table 4.** Results from bivariable and multivariable logistic regression models examining predictors of being at risk of anemia among Malaysian children aged ≥6 months to ≤36 months.

Variable	Crude odds ratio (95% CI)	*P *value	Adjusted odds ratio (95% CI)	*P *value
Sex				
Female	1		1	
Male	0.98 (0.77‐1.25)	.876	0.96 (0.75‐1.23)	.74
Age				
>12‐36 months	1		1	
6‐12 months	1.63 (1.27‐2.09)	<.001	1.73 (1.34‐2.24)	<.001
Race				
Asian, Chinese	1		1	
Asian, Bumiputera	1.47 (0.92‐2.35)	.104	1.34 (0.80‐2.24)	.25
Asian, Indian	0.73 (0.39‐1.30)	.298	0.72 (0.38‐1.31)	.29
Asian, Malay	1.42 (1.04‐1.95)	.029	1.23 (0.88‐1.73)	.22
Residence location				
Rural	1		1	
Urban	0.85 (0.66‐1.09)	.195	0.96 (0.72‐1.27)	.76
Education				
Bachelor’s degree or higher	1		1	
Secondary education and below	0.96 (0.72‐1.27)	.754	0.84 (0.62‐1.14)	.25
Household income				
Low	1		1	
Middle or high	0.81 (0.63‐1.03)	.086	0.78 (0.59‐1.02)	.07
Physical activity level				
Sedentary or lightly active	1		1	
Moderately active or vigorously active	1.04 (0.74‐1.50)	.812	1.19 (0.80‐1.79)	.39
Gestational age at birth				
Term	1		1	
Preterm	0.97 (0.72‐1.29)	.839	0.98 (0.73‐1.32)	.91
Mode of delivery				
Vaginal	1		1	
Cesarean	0.75 (0.57‐0.99)	.046	0.78 (0.58‐1.04)	.09
Birth order				
First	1		1	
Second or more	1.23 (0.93‐1.63)	.144	1.11 (0.83‐1.49)	.49
Family history of thalassemia				
No	1		1	
Yes	1.15 (0.16‐5.92)	.872	1.26 (0.17‐6.90)	.79
Family history of anemia				
Yes	1		1	
Not known	0.89 (0.60‐1.34)	.573	0.95 (0.62‐1.49)	.83
Weight-for-age Z score	0.91 (0.82‐1.00)	.046	0.88 (0.80‐0.98)	.02
Length-for-age Z score	0.95 (0.88‐1.03)	.206	0.74 (0.41‐1.31)	.31
Weight-for-length Z score	0.95 (0.85‐1.05)	.313	0.65 (0.28‐1.47)	.30

## Discussion

### Principal Findings and Comparison with Previous Works

The study assessed the prevalence and profiles of Malaysian children aged ≥6 to ≤36 months being at risk of anemia using a noninvasive Hb approach (the Masimo Rad-67 Pulse CO-Oximeter). Unlike the conventional methods of assessing anemia, this noninvasive screening approach offers rapid, accurate, and reproducible results, favoring the implementation in maternal child health clinics for early detection and prompt treatment before the condition worsens to severe anemia-related complications. This study had a response rate of 97.9% and only 7 out of 1227 patients were not cooperative to be included in the study. A high response rate among participants to be part of the study indicates their receptiveness to a noninvasive approach. The lack of parental consent due to invasive approaches has been observed and highlighted as a limitation in a similar study [21]. Caregivers of children highly favor noninvasive disease screening methods that lead to better testing compliance compared to invasive techniques that can cause discomfort and pain [22]. Noninvasive methods also reduce the risk of infection and could potentially be more cost-effective due to less usage of consumables and reagents [23].

This study found that 30% of Malaysian children aged ≥6 to ≤36 months were at risk of anemia. The observed prevalence was slightly higher than the 25% national prevalence of anemia among children below 5 years [19]. Moreover, the observation was also higher than that observed in two previous studies, which reported prevalence rates of 21.2% [23] and 22.3% [24], respectively. However, the observed prevalence was lower than the prevalence rate of 42.4% reported by Sabri et al [12] and 46.5% reported by the 2022 National Health and Morbidity survey [7]. As a screening tool for anemia, the Masimo Rad-67 Pulse CO-Oximeter is less stringent, which may account for the slightly higher prevalence observed in this sample population. As this is as a screening tool, confirmatory laboratory tests will be required for a diagnosis after screening.

This study focused on looking at contributing factors of anemia, although it did not measure iron content to evaluate the prevalence of IDA. A retrospective study in Pulau Pinang showed that the rate of IDA was 24% among children [24]. However, the 24-hour dietary recall that was performed may give an idea of the children’s iron intake. It will be interesting to see if the children’s intake of iron and other nutrients has any association with anemia development in this study. In this study, parental interpretation was used to measure physical activity. No specific children’s physical activity measurement tool was used.

Although several measures have been undertaken, the observed prevalence of being at risk of anemia in Malaysia is substantially high, implying a need for more focused interventions aimed at prevention, early screening, and detection as well as treatment. In this regard, the extensive use and adoption of noninvasive screening tools at maternal and health clinics, which are points of child vaccination and regular health checks, would be essential for the early detection and providing prompt treatment, thus curbing the adverse complications of anemia in children [25-27].

The study explored factors associated with anemia among Malaysian children, of which age and weight-for-age were significant. The study results indicate that a higher risk of anemia is more common among children aged 6‐12 months and lower weight-for-age. As expected, our results partly agree with the existing literature that has also reported children 6‐12 months to have a higher risk of anemia compared to older children, due to increased iron needs that, if not provided by weaning foods, put this age group at a higher risk of serious anemia [3,4,28]. This aligns with previous studies that have reported a similar trend in India [29], Ethiopia [30], Brazil [31], and Peru [32].

Notably, although nonsignificant on adjusted regression analysis, children of the Asian-Malay race showed higher proportions of being at risk of anemia (65%) than other races, a finding that aligns with previous studies that have reported racial disparities in the risk of anemia [33,34]. Khalil et al showed a vast difference in the prevalence of anemia between the Orang Asli tribes in Malaysia, which was attributed to differences in socioeconomic background and other risk factors of anemia [35]. Similar to another study, anemia prevalence differences observed among racial and ethnic groups in this study can also be due to different food practices and low dietary intake of iron [21].

We expected thalassemia, a genetic predisposing factor affecting one’s Hb concentration, to be positively associated with the risk of anemia, which is in contrast to this study’s finding. In our study, Thalassemia status is based on parental and carer’s reporting and could be subjected to reporting error, since as high as 26.3% of the subjects were unaware of their thalassemia status. Similarly, cesarean section as a mode of delivery is reported to have a negative impact on child feeding practices compared to normal vaginal delivery [21]; thus, we expected a positive association with anemia risk, which also deviates from our finding. Nonetheless, the difference in sample size and composition in our study could explain the observed mismatch. Additionally, mothers with children born of cesarean section and with thalassemia may be more aware of the increased risk of anemia from clinical counseling and education [36]. Therefore, they may be more likely to take extra measures to prevent anemia in their children, unlike mothers with no such known risk, which may explain our finding of surprisingly increased risk of anemia among children born via normal vaginal delivery and with no family history of thalassemia.

Malnutrition has been previously recognized as a factor for anemia among underweight children [28,37]. Additionally, micronutrient deficiency, food insecurity, and poverty have been established as factors leading to underweight children [28,38]. A study in China found that malnutrition resulted from the caregiver’s lack of knowledge of child feeding practices [37], which may also be a contributing factor to anemia among children in this study. In addition, although the current data revealed that children from lower-income families had a higher proportion of being at risk of anemia, the household income surprisingly showed no statistically significant association with being at risk of anemia in the previous study [37].

It is worth noting that the observed high prevalence of anemia risk among children, including Malaysian children, is driven by complex interlinked factors including socioeconomic status, access to healthcare, dietary patterns, and cultural practices, amongst others [21,28,37,38]. A few such factors have been re-echoed in this study. The socioeconomic status, for example, affects food access and dietary quality, which are primary causes of anemia [28,38]. The socioeconomic status also affects healthcare access, without which the impact of anemia among children may be worsened. This calls for a comprehensive and inclusive approach to designing anemia prevention programs, considering the interaction of various key drivers.

### Implications of Study Findings

Our study findings have some practical implications for addressing the observed high risk of anemia among Malaysian children. There is a need for more efforts in strengthening prompt community screening of anemia nationwide to enable the early detection and, thus, appropriate intervention or treatment, and this could be achieved by the adoption and use of noninvasive screening tools like the Masimo Rad-67 Pulse CO-Oximeter. The study results also imply that the Masimo Rad-67 Pulse CO-Oximeter could be a useful tool in the clinical assessment of Malaysian children for anemia, which could be incorporated into regular child immunization and monitoring visits. These efforts and other anemia prevention strategies should focus on children aged 6‐12 months of Asian Malay heritage or race, and underweight children because they have a higher risk of anemia. Moreover, more efforts are needed in community education and sensitization about anemia risk, its consequences and prevention, and this should focus on the mothers and caregivers of children under 5 years. Additionally, nutritional education should be incorporated in the discussions with parents and caregivers after the examination of infants and young children to reduce the incidence of being underweight among children and to ensure anemia prevention and reduction.

### Limitations

The study has some limitations. Some of the data, especially background predictor variables, were based on self-reporting, risk recall, and misclassification biases. Moreover, given the cross-sectional design of the study, no causal inference can be drawn between anemia and the other factors considered, beyond mere associations. Despite the limitations, the study provides valuable information on the prevalence of the risk of anemia and associated factors among Malaysian children using a noninvasive assessment tool.

### Conclusions

Using a noninvasive screening tool, the study found that 30% of Malaysian children aged ≥6 to ≤36 months are at risk of anemia. Moreover, children aged 6‐12 months and of Asian-Malay heritage or race had high odds of anemia. Weight-for-age was also negatively associated with the risk of anemia. Therefore, there is a need for more efforts in targeted community screening to enable the early detection and prompt treatment of anemia. More community education and awareness about anemia, including nutrition education, is also needed to address the high levels of anemia in the country.

## Abbreviations

Hb: hemoglobin
IDA: iron deficiency anemia
